# Dying of VOC-202012/01 — multimodal investigations in a death case of the SARS-CoV-2 variant

**DOI:** 10.1007/s00414-021-02618-8

**Published:** 2021-06-05

**Authors:** Fabian Heinrich, Carina Romich, Tamara Zimmermann, Inga Kniep, Antonia Fitzek, Stefan Steurer, Markus Glatzel, Dominik Nörz, Thomas Günther, Manja Czech-Sioli, Nicole Fischer, Adam Grundhoff, Marc Lütgehetmann, Benjamin Ondruschka

**Affiliations:** 1 Institute of Legal Medicine, University Medical Center Hamburg-Eppendorf, Butenfeld 34, 22529 Hamburg, Germany; 2 Institute of Pathology, University Medical Center Hamburg-Eppendorf, Hamburg, Germany; 3 Institute of Neuropathology, University Medical Center Hamburg-Eppendorf, Hamburg, Germany; 4 Institute of Medical Microbiology, Virology and Hygiene, University Medical Center Hamburg-Eppendorf, Hamburg, Germany; 5 Heinrich-Pette-Institute, Leibniz-Institute for Experimental Virology, Hamburg, Germany

**Keywords:** SARS-CoV-2, Variant of Concern-202012/01, B.1.1.7, 501Y.V1, Postmortem, Coronavirus-disease-19, Respiratory infections

## Abstract

The current pandemic with *Severe acute respiratory syndrome-coronavirus-2* has been taking on new dynamics since the emergence of new variants last fall, some of them spreading more rapidly. Many countries currently find themselves in a race to ramp up vaccination strategies that have been initiated and a possible third wave of the pandemic from new variants, such as the *Variant of Concern-202012/01* from the *B.1.1.7* lineage. Until today, many investigations in death cases of Coronavirus-disease-19 have been conducted, revealing pulmonary damage to be the predominant feature of the disease. Thereby, different degrees of macroscopic and microscopic lung damage have been reported, most of them resembling an Acute Respiratory Distress Syndrome. Far more, systemic complications of the disease such as pulmonary embolisms have been described. However, neither morphologic nor virologic findings of patients dying of the new variants have yet been reported. Here, we report on a comprehensive analysis of radiologic, morphologic, and virologic findings in a fatal case of this variant.

## Introduction

On March 12, 2020, the World Health Organization declared *Severe acute respiratory syndrome-coronavirus-2* (*SARS-CoV-2*) and Coronavirus-disease-19 (COVID-19) to be a pandemic [1]. Since 2.5 million people died of the novel pathogen, and case fatality rates ranging from 0.7 to 7.2% have been reported [2–4]. Fatal outcomes of the disease were mainly attributed to respiratory failure by Acute Respiratory Distress Syndrome (ARDS) [5]. In line with this, different degrees of macroscopic and microscopic lung damage have been described in deceased of COVID-19 [6–8]. In particular, the microscopic damage constitutes diffuse alveolar damage with hyaline membranes. Simultaneously, systemic complications of the disease were suggested at an early stage of the pandemic: Coagulopathy and thromboembolic complications have consistently been reported in COVID-19 patients alive and death [9, 10].

While the characteristics and pathomechanisms of the disease are becoming more and more understood, new variants of *SARS-CoV-2* are emerging all over the world [11]. One of these emerged in the Region of Kent (United Kingdom) and is defined by 23 characteristic mutations [12]. This new variant gained international attention when epidemiologic data on spread dynamics were first published [13]. Preliminary data suggest this variant to spread more effectively compared to previously circulating lineages [13]. An explanation could be a mutation, N501Y, in the receptor-binding domain of the spike protein, which is important for cellular entry [14]. Other explanations include prolonged viral shedding [15]. Therefore, and due to the possibility of higher mortality rates reported in preliminary reports, concerns have been raised on this new variant [16].

Further analyses successfully confirmed the new variant to spread more effectively [17]. Whether the course of the disease is more severe and ultimately deadly remains to be determined. As of today, autopsy data on disease-specific findings in patients dying of the new variant are missing. Here, we report on detailed radiologic, morphologic, and virologic findings in a fatal case of this new variant (*B.1.1.7*/*Variant-of-concern-202012*/*01* [*VOC-202012/01*]).

## Methods

### Postmortem computed tomography

Philips Brilliance 16-slice MDCT was used for a whole-body scan (slice thickness 1 mm, Pitch 1.5, 120 kV, 230–250 mAs). The thorax was additionally scanned using higher resolutions (slice thickness 0.8 mm, Pitch 1.0, 120 kV, 230–250 mAs).

### External and internal examination

External and internal corpse examination were performed in accordance with guidelines on the forensic full postmortem examination by the German Society of Legal Medicine (AWMF 054/001) [18]. Special consideration of recent guidelines on the handling of COVID-19 deaths has been made [19]. Photographic and written documentation was performed thoroughly.

### Histology

Tissues were fixed in formalin for 24 h and subsequently embedded in paraffin. Hereafter, hematoxylin and eosin (H.&E.) stainings were performed using a Ventana Benchmark XT automated staining system. Additionally, immunohistochemical stainings were performed for B- and T-lymphocytes in the lungs, as well as for activated astrocytes, and activated microglia in the frontal cortex using antibodies directed against the human glial fibrillary acidic protein (GFAP) (clone 6F2; Dako, Glostrup, Denmark; dilution 1:200) and HLA-DR (clone CR3/43; Dako, Glostrup, Denmark; dilution 1:200). Slides were examined by experienced pathologists and neuropathologists (S.S. and M.G.) and were electronically scanned at high magnification (× 40) as high-resolution images (1900 × 1200 pixels) with a NanoZoomer 2.0-HT (Hamamatsu Photonics, Hamamatsu, Japan).

### Virology

#### RT-PCR for SARS-CoV-2

Nasopharyngeal swabs withdrawal and analysis for *SARS-CoV-2* RNA were performed as recently described [20]. Reverse-transcription quantitative polymerase chain reaction (RT-qPCR) for N501Y and del HV69/70 as described elsewhere in a combination with commercially available assays screening for E484K and P681H (TIB Molbiol, Berlin, Germany) as recommended by the manufacturer was additionally done to screen for the *VOC-202012/01* lineage [21].

For further analysis, native tissue samples and bodily fluids were obtained during the conventional autopsy. Tissue samples of 1 cm^3^ were taken using different scalpels and petri-dishes and bodily fluids by using different syringes and cannulas. The specimens were immediately processed and analyzed for the E-gene of *SARS-CoV-2* by RT-qPCR [22]. For quantification, standard in vitro-transcribed RNA of the E gene of *SARS-CoV-2* was used. The human ß-globin gene was quantified by PCR (Life technologies, Thermo Fischer, USA) and used for normalization of viral RNA loads.

#### SARS-CoV-2 whole-genome sequencing and bioinformatics analysis

Viral genome sequencing was carried out as recently published [23, 24] with the following modifications: Amplicon sequencing and sequencing library generation were performed using the CleanPlex SARS-CoV-2 Panel (Paragon Genomics, CA, USA). Samples were sequenced on Illumina MiSeq using a 300 cycle MiSeq v2 reagent kit (Illumina, CA, USA). Quality-filtered paired-end amplicon sequencing reads were trimmed for Illumina sequencing adapters as well as correct amplicon primer pair sequences. Reads were then merged and subsequently aligned to *NC_045512.2* using minimap2 [25] with default settings for short read alignment. Variants were called using freebayes Bayesian haplotype caller v 1.3.1 [26] with ploidy and haplotype-independent detection parameters to generate frequency-based calls for all variants passing input thresholds (-K -F 0.5). Input thresholds were set to at least 10 variant supporting reads with a minimum base quality of 30 (-C10 -q30). Only high confidence variants present in ≥ 50% of reads were included and annotated using ANNOVAR [27].

### Case report

We report on a 70-year-old woman who presented to the emergency service in an unconsciousness, asystole state. Cardiopulmonary resuscitation was performed using manual chest compression and high doses of catecholamines without return of spontaneous circulation. Then, the patient was declared dead. The onsite case evaluation revealed that the patient got up confused in the middle of the night and has fallen to the floor unconscious. Her husband reported no other symptoms prior to death. Only routine RT-qPCR screenings at the Institute of Legal Medicine revealed an infection with the novel variant of *SARS-CoV-2*. In accordance with the German infection protection law, the public health authorities were informed immediately.

The retrospective case evaluation by the emergency services, public health authorities, and the Institute of Legal Medicine revealed that the woman in fact became to feel sick 3 days prior to death. She started coughing, and the following night, she developed a fever with heavy sweating. No myalgia, headache, or dyspnea was reported. Her general condition continued to deteriorate until she presents unconscious. However, no possible transmission event could be evaluated.

Individual risk factors were evaluated from the patient’s medical records. Relevant pre-existing diseases were arterial hypertension, bronchial asthma, coronary heart disease, and chronic kidney disease. Furthermore, an adrenal adenoma was reported.

### Postmortem computed tomography

The lungs showed bilateral diffuse to global consolidation with a focus to the centrilobular areas. Reticular changes with peripheral accentuation can be found in both lungs.

Also, scattered ground glass opacities were found with a focus to the dorsal lung areas. In accordance, with resuscitation procedures, serial rib fractures were found. Additionally, general and coronary atherosclerosis to moderate degrees, cardiac hypertrophy, and minutely atrophic kidneys were detected.

### Findings of the autopsy and histology

The 70-year-old woman presents over-weighted (BMI: 29.5 kg/m^2^) without external or internal signs of trauma despite chest compression-related rib fractures and bleedings.

**Fig. 1:**
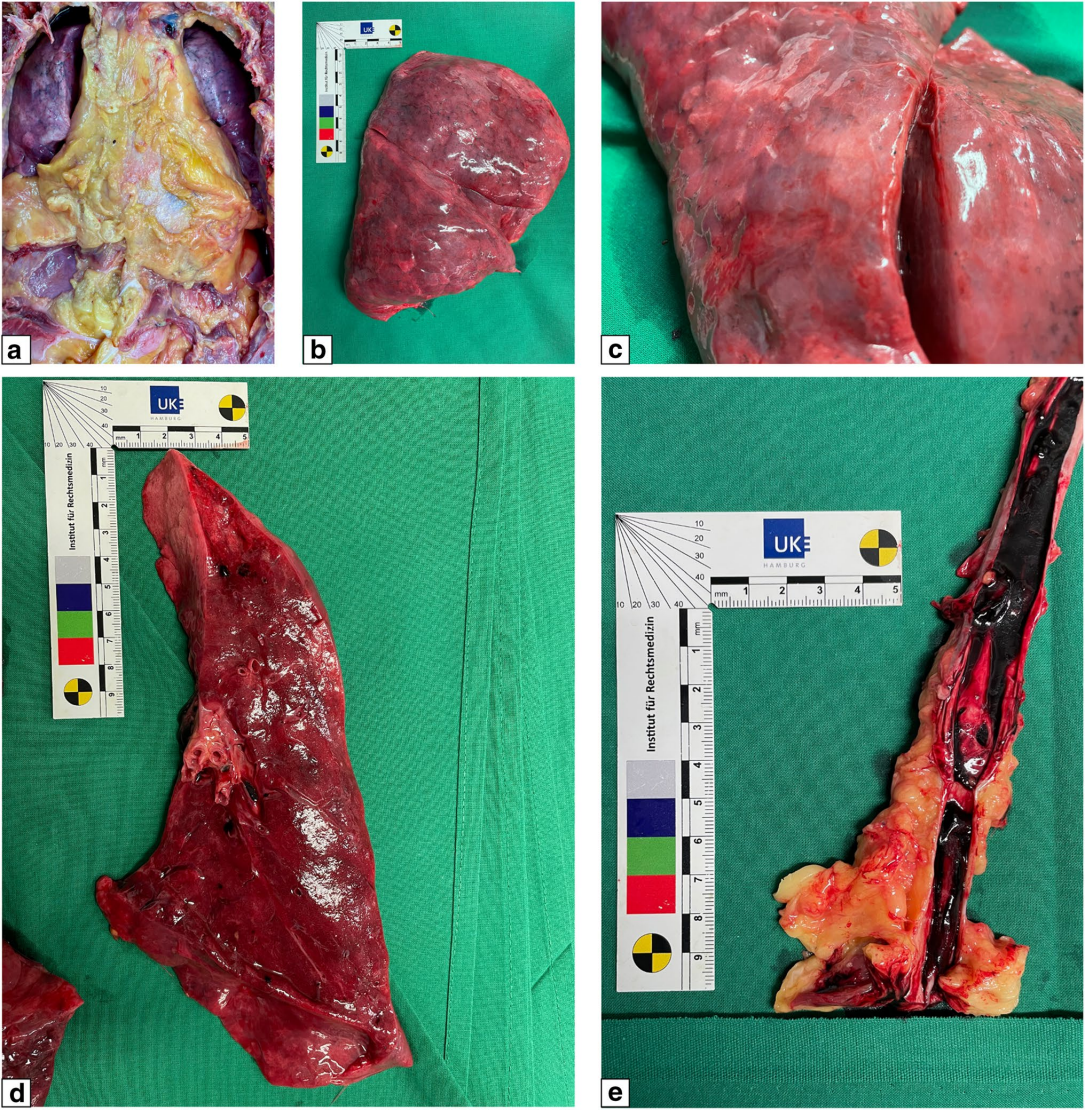
Macroscopic findings during the autopsy. An overview of the chest cavity (**a**) and more detailed pictures of the surface and cross-sections of the lungs (**b**–**d**) illustrating bilateral deep red hyperemic discolorations with peripheral hyper-inflation of the lungs. Exemplary deep venous thromboses of the femoral vein are illustrated (**e**)

**Fig. 2:**
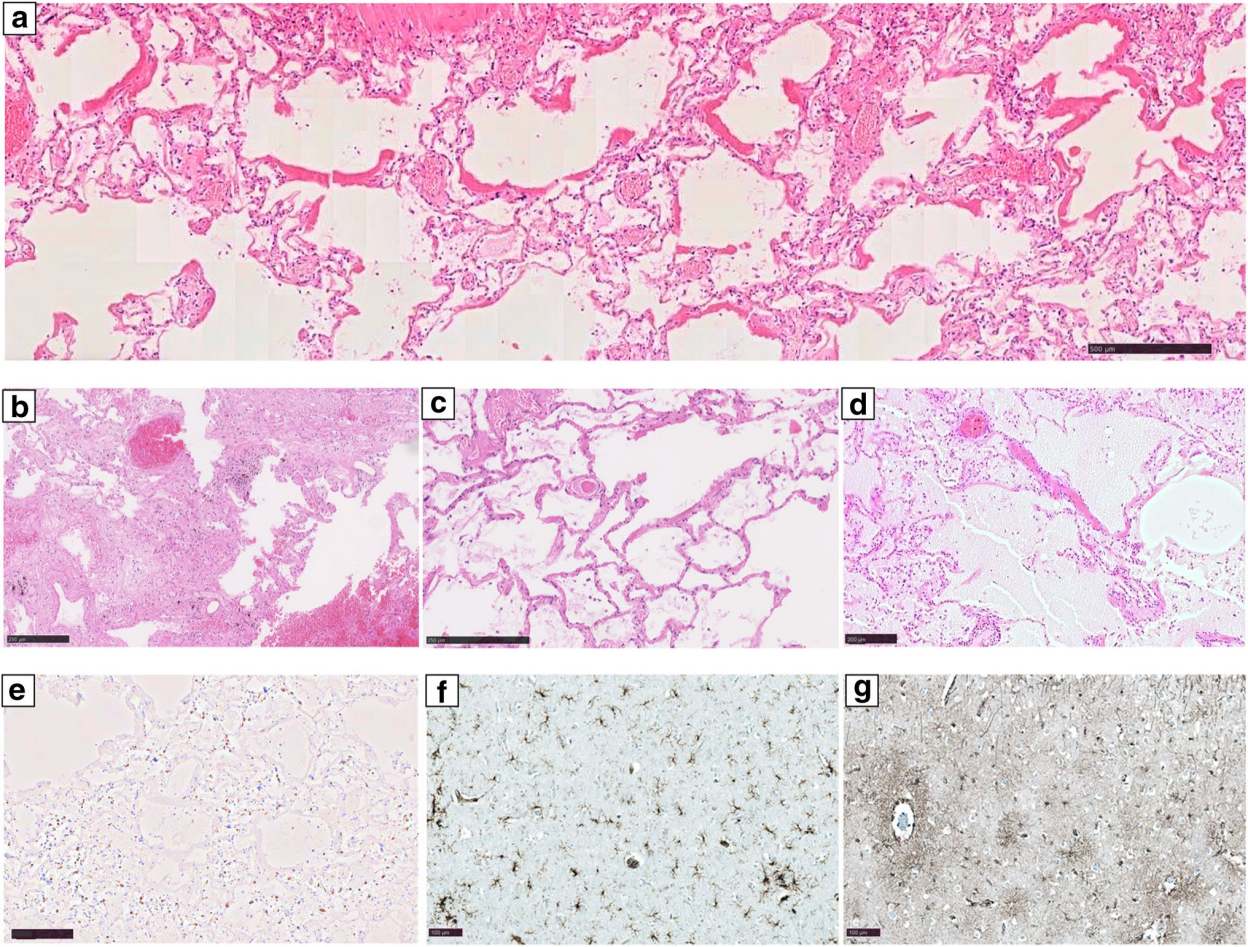
Microscopic findings of the histologic examination. The lungs of the deceased showed signs of diffuse alveolar damage with hyaline membranes (**a**), hemorrhagic interstitial and alveolar infiltration (**b**), the presence of microthrombi (**c**), and an intra-alveolar oedema (**d**) (H.&E.). Interstitial accumulation of CD8 positive cells (anti-CD8 antibody) was found in the central areas of both lungs (**e**). Perivascular astrogliosis (anti-glial fibrillary acidic protein antibody) and mild-to-moderate activation of microglia, with occasional microglial nodules in the frontal cortex (anti-HLA-DR antibody) (**f**–**g**). Picture specific scale bars are shown

A full autopsy has been performed. Examining the lungs, we found hyperaemic areas of more dense consistency with a particular focus on the centrilobular regions of both lungs and some subpleural hemorrhages (Fig. 1a–d). Minimal pleural effusions (right side: 50 ml, left side: 50 ml) and an intra-alveolar lung oedema accompanied the observed inflammatory processes (Fig. 2d, e). The latter resulted in a lung weight of 1500 g in total (right lung lobe: 855 g, left lung lobe: 645 g). Detailed histologic examination revealed inflammatory changes with a focus on the central areas of both lungs. More precisely, lymphocytic cell infiltration predominantly consisting of CD8 positive cells was found within the interstitial lung areas (Fig. 2e). Local aggregation of CD8 positive cells within the alveoli was found. Disseminated spots of activated type II pneumocytes were observed, and hyaline membranes and the presence of microthrombi within the lungs accompany the reported findings (Fig. 2a, c). Also, hemorrhagic alveolar infiltration was found (Fig. 2b), and mediastinal lymph nodes showed reactive hyperplasia. Concurring inflammatory changes in the lungs, morphological signs of systemic inflammation were found in terms of “septic spleen” and lipid depletion of the adrenal glands.

Interestingly, we also found peripheral pulmonary embolisms (PE) involving the segmental (in the left pulmonary artery flow area) and sub-segmental vessels (in the right pulmonary artery flow area) in this case. The latter presented slightly ribbed without adhering to the vessel wall. Accordingly, deep vein thrombosis (DVT) in the leg veins was found without adherence of the thrombi to the vessel walls (Fig. 1e). The blood in the heart chambers and large vessels presented fluid.

Neuropathologic examinations of the brain showed astrogliosis pronounced around vessels and mild-to-moderate activation of microglia, with occasional microglial nodules (Fig. 2f–g). There were no signs of fulminant potentially virus-induced encephalitis, and there was no evidence of cerebral bleeding or small-vessel thromboses.

Apart from the signs of the diseases previously diagnosed, other organs showed no significant macroscopic or microscopic changes.

### Virologic examination

The initial virologic assessment revealed high nasopharyngeal loads of the *SARS-CoV-2* E-gene with 3.3 × 10^8^ RNA copies/ml. Typing assays for *VOC-202012/01* revealed positive results for N501Y and P681H but not for E484K, thereby successfully confirming the typical characteristics of *VOC-202012/01*.

**Fig. 3:**
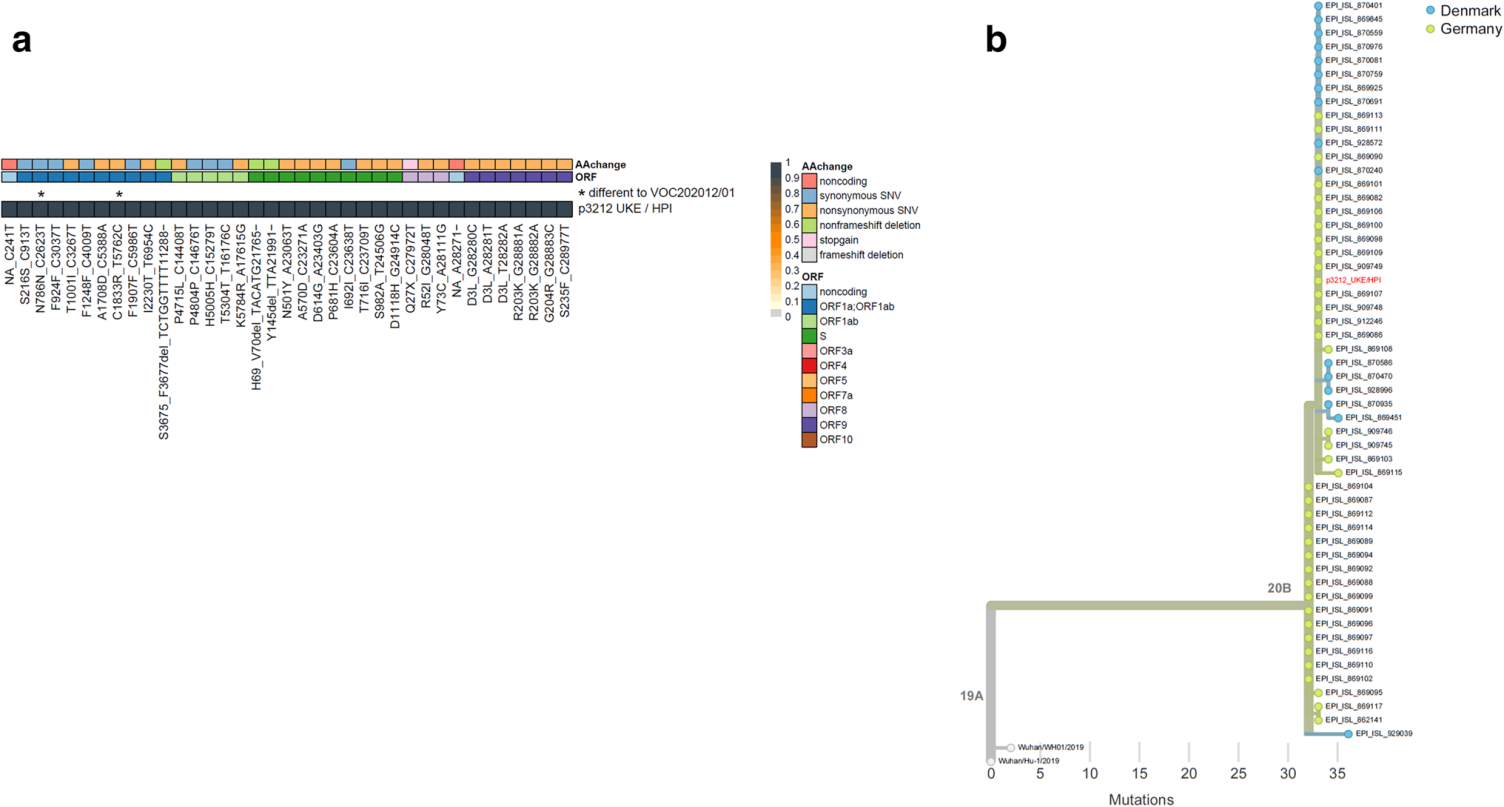
SARS-CoV-2 whole genome sequencing. **a** The heat map shows the position (nucleotide positions and amino acid, aa, positions are shown below), identity (indicated with a color code for the individual orfs, synonymous aa substitutions are shown in blue, non-synonymous aa substitutions in orange), and frequency (from 0, gray to 100% frequency, dark blue) of variant nucleotide positions detected by SARS-CoV-2 full genome. Variant sequences of the sample p3212_UKE/HPI are given relative to the set of SNPs identified in B.1.1.7 as previously described (https://virological.org/t/preliminary-genomic-characterisation-of-an-emergent-sars-cov-2-lineage-in-the-uk-defined-by-a-novel-set-of-spike-mutations/563). Additional variant positions present in p3212_UKE/HPI are marked with an asterisk. **b** Phylogenetic analysis of p3212_UKE/HPI sample within the context of B.1.1.7 isolates. Samples were analyzed and visualized within the phylogenetic context by next strain (nextstrain.org) using data available through GISAID (gisaid.org); GISAID identifiers are indicated at the right. Shown are only closely related sequences with 4 nucleotide substitution difference from the sequence p3212_UKE/HPI (in red). Sequences sampled in Germany, Schleswig–Holstein, are shown in green, while sequences from Denmark are in blue

These results were underlined by the results of amplicon sequencing; thereby confirming the assignment to the *B.1.1.7* pangolin lineage with its typical deletions and point mutations in the S-gene and other viral genome regions (Fig. 3a). In addition to the described core mutations of *VOC-202012/01*, the sequence contains two mutations in orf1ab: one synonymous mutation in the nsp2 gene, nucleotide (nt) position 2623, and one non-synonymous mutation in the nsp3 gene, encoding the papain like protease. The single nucleotide polymorphism (SNP) at nt 5762, results in an amino acid (aa) change at position 1833 from cysteine to the basic aa arginine. This genotype has been described previously in sequences entered in the GISAID database [28] sampled in Germany, Schleswig–Holstein, and Denmark (Fig. 3b).

**Fig. 4:**
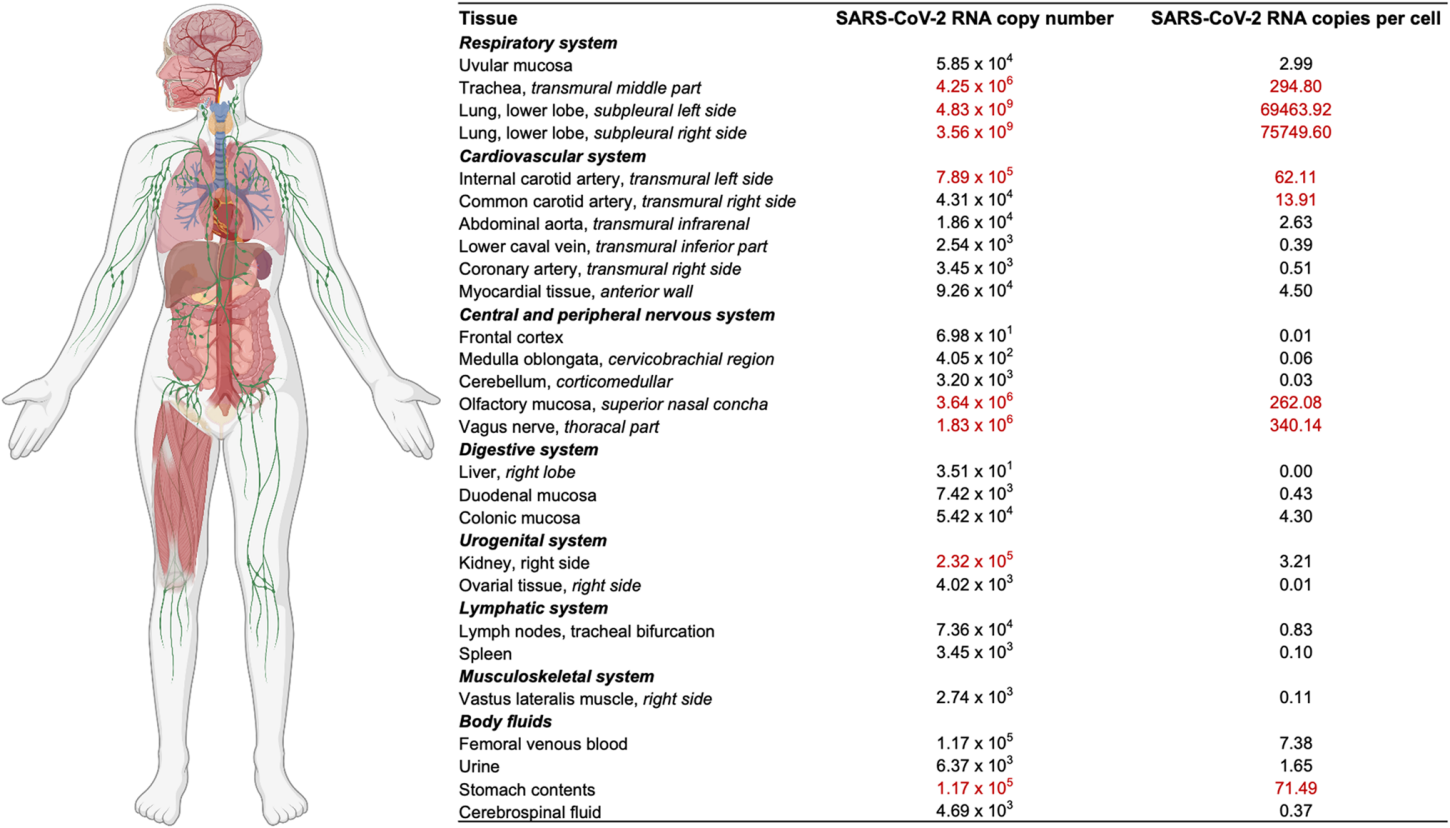
Distribution of *SARS-CoV-2* RNA loads in different tissues and bodily fluids. Real time-quantitative polymerase chain reaction has been performed from separately taken tissues and bodily fluids. Quantified *SARS-CoV-2* RNA copy numbers (E-gene) are shown per milliliter and normalized per cell. Viral copy numbers exceeding those of blood are marked in red. This figure was created with BioRender.com

Furthermore, we examined the presence of *SARS-CoV-2* RNA in a comprehensive collection of different tissues and bodily fluids (Fig. 4). Thereby, viral RNA was detected in virtually all tissues and bodily fluids analyzed. In femoral venous blood, 1.17 × 10^5^ *SARS-CoV-2* RNA copies per milliliter were detected. Titres exceeding the viral load in the blood are marked in red (Fig. 4).

Matching high viral titres in the nasopharynx, we found high viral loads in the upper respiratory tract (i.e., olfactory mucosa, and trachea) and both lung lobes (Fig. 4). Also, stomach contents were tested highly positive for *SARS-CoV-2* RNA (71.49 copies/cell). Of note, not only the vagus nerve but also the common carotid artery being part of the para-pharyngeal space show high amounts of *SARS-CoV-2* RNA (N. vagus: 340.14 copies/cell, A. carotis communis: 13.91 copies/cell). In the bodily fluids tested, such as urine and cerebrospinal fluid (CSF), low viral loads were shown (urine: 1.65 copies/cell, CSF: 0.37 copies/cell).

## Discussion

Here, we report on a 70-year-old woman dying of COVID-19 due to a new variant of *SARS-CoV-2* (*B.1.1.7*/*VOC-202012/01*).

In the postmortem computed tomography, ground glass opacities and reticular changes of both lungs have been described as being COVID-19-associated lung changes [29]. These are also the radiological characteristics that we were able to identify in this case. However, the observed radiologic findings are reported to be not disease-specific [29]. Apart from previously described changes in the literature, no other pulmonary radiologic features were observed [29].

In line with recent reports, hemorrhagic alveolar infiltration was found being a typical autoptic sign of viral pneumonia [30]. Far more, macroscopic findings of heavy lungs and microscopic findings of intra-alveolar oedema, hyaline membranes, and microthrombi quite appropriately illustrate the early stages undergoing an Acute Respiratory Distress Syndrome (ARDS) as frequently reported in COVID-19 patients [6–8]. Aggravated by peripheral pulmonary embolisms, an impaired gas exchange with global respiratory insufficiency due to COVID-19 has been the most probable cause of death in this case.

Coagulopathies and thromboembolic complications to occur in COVID-19 patients are a topic of ongoing investigations [9, 31, 32]. Already the first reports described mild-to-severe coagulation impairments in COVID-19 patients [10]. Concurring these results, first autopsy studies revealed increased frequencies of pulmonary embolisms and deep vein thrombosis in 33 and 57% of the cases, respectively [9]. Subsequent case series by Edler et al. also successfully confirmed these results [33]. In line with such findings, also in this case, PE and DVT have been detected. Risk factors for the development of severe coagulation disorders and thromboembolic complication in COVID-19 patients and the respective pathomechanisms remain elusive until today.

Neuropathologic alterations seen in this patient are in line with published data [34, 35]. Also, here, there were no signs of fulminant virus-induced encephalitis, and the perivascular pronounced activation of the neuroimmune system supports the view that disease-associated neuroinflammation may contribute to neurologic deficits seen in COVID-19 patients [36].

The other macroscopic and microscopic findings observed represent changes associated with the patient’s pre-existing medical conditions and older age. Interestingly, most of the pre-existing conditions, namely arterial hypertension, coronary heart disease, and chronic kidney disease, have been shown to be associated with severe and/or fatal courses of COVID-19 [37]. Thus, the patient was particularly susceptible to a severe and fatal course of the disease. Beyond that, all virus-related macroscopic and microscopic findings are similar to those of several reports on autopsy findings in COVID-19 decedents from a variety of authors all over the world. No surprising potentially lineage-specific morphologic findings were documented.

Virologic examinations reveal high viral loads in the nasopharynx of the deceased, indicative of an acute course of COVID-19 [38]. The trachea and lungs of the deceased were also highly positive, matching the pulmonary tropism of the virus. In line with the latest findings of Meinhardt et al., the olfactory mucosa was also affected by the virus [39]. Positive stomach contents could be explained by ingestion of virus particles into the stomach. An explanation for the highly positive testing of the vagus nerve and carotid artery could result from the spatial proximity to the pharyngeal space. Also known as “Danger space,” bacterial spread has been described in these regions [40]. It is worth noting that all tissues tested were positive for *SARS-CoV-2* RNA. While cross-contamination during the autopsy was excluded by separate surgical instruments, dissemination of viral RNA by the patient’s viremia cannot be excluded. Whether tissues with RNA levels exceeding those of blood represent sites of active viral replication or accumulation of viral RNA needs further studies. It was shown that the N501Y mutation leads to an increased affinity to the ACE2 receptor. This might lead to an altered organtropism. However, the RNA tropisms observed here do not differ to the N501 wildtype. Further studies are needed to find potential correlates of increased mortality, i.e., altered or enhanced organ tropisms.

Important to mention here is the added value of postmortem screenings for *SARS-CoV-2*. At the point of death of the patient, first reports on the new variant in Germany have been made [41]. Ad-hoc analyses by the Robert Koch Institute from the week after this death case revealed *B.1.1.7* positivity in 5.8% of all nationwide screened cases [42]. The incidental finding of *SARS-CoV-2* in this case, e.g., might be indicative of a previously unknown cluster of *VOC-202012/01* infections. Following this insight, the health authorities have traced contacts of all persons involved and ordered a quarantine. Therefore, this case not only underlines the importance of such screenings for questions relevant to occupational safety but also emphasizes its importance for infection control purposes. The potential of next-generation sequencing in postmortem applications is highlighted.

## Data Availability

Not applicable.
